# Complex Neurochemical Microstructure of the Stria Terminalis in Infant and Adult Macaque Monkey

**DOI:** 10.3389/fnana.2022.891608

**Published:** 2022-05-25

**Authors:** Mitali Sakharkar, Kathleen S. Rockland, Alvaro Duque

**Affiliations:** ^1^Boston University School of Medicine, Boston, MA, United States; ^2^Department of Anatomy and Neurobiology, Boston University School of Medicine, Boston, MA, United States; ^3^Department of Neuroscience, Yale University School of Medicine, New Haven, CT, United States

**Keywords:** amygdala, anterior commissure, bed nucleus, extended amygdala, interstitial neurons, myelin, tractography

## Abstract

The stria terminalis (ST) is a major bidirectional fiber tract anchored in the amygdala and bed nucleus (BNST). Extensive investigations in rodents report a complex arrangement of neurochemically diverse neurons within the ST, but fewer data are available for non-human primates. Given the functional importance of the ST, we investigated its microarchitecture in one newborn, four infant, and two adult macaque brains, by parallel immunocytochemical series for cells or fibers. Main results are as follows: (1) The pan-neuronal marker NeuN shows scattered neurons and small neuronal clusters in both the dorsal and ventral ST, but more numerous dorsally; (2) smaller neuronal subpopulations are labeled by calretinin (CR), neuropeptide Y (NPY), calbindin (CB), and somatostatin (SOM), of which the CR + neurons are the most numerous; (3) the infant brains on average have more neurons in the ST than the adult brains, but across our sample, there is notable individual variability; and (4) fiber architectonics have a complex organization, which can be referenced to myelin-poor or myelin-dense zones. Myelin-poor zones coincide with concentrations of fibers positive for CB, CR, or tyrosine hydroxylase (TH). Neurons have been reported in other white matter domains (e.g., anterior commissure, corpus callosum, cingulum bundle, and subcortical white matter). Like these, at least some neurons within the ST may give rise to long-distance connections, and/or participate in more local functions, such as vascular regulation or axon guidance/maintenance.

## Introduction

The stria terminalis (ST) is a major fiber tract associated with the amygdala and bed nucleus of the ST (BNST) and is conserved across human, non-human primates (NHP), rodents, and other species (Johnston, 1923; Novotny, 1977; Strenge et al., 1977; Martin et al., 1991). In primates, it is clearly identified as a C-shaped tract, medial to the tail and body of the caudate nucleus for much of its extent. In part because of this characteristic location, the three-dimensional trajectory has been extensively investigated (Vasung et al., 2010; Mori et al., 2017; Oler et al., 2017). In contrast, the finer organization of subpopulations of fibers and their general orientation has been relatively under-investigated in humans and NHP [e.g., Martin et al. (1991)], especially in comparison with multiple excellent studies in rodents (Alheid et al., 1998; Shammah-Lagnado et al., 2000; McDonald, 2003). Moreover, anatomical studies, also mainly in rodents, have identified a complex organization of neurons embedded in the ST, which has been discussed as a component of the “extended amygdala” (de Olmos and Heimer, 1999).

In this report, we take advantage of a recently available database (MacBrainResource, MBR) to investigate the complex neurochemical microstructure of the ST in newborn (PD7), infant, and adult macaque brains. In the first section, we describe neuronal populations, visualized in total by NeuN and, as subpopulations, by antibodies against calbindin (CB), calretinin (CR), neuropeptide Y (NPY), and somatostatin (SOM). In the second section, we focus on several neurochemically defined fiber sub-bundles and their spatial patterns of overlap or complementarity within the ST. Bundles are delineated on the basis of fibers reactive and non-reactive to antibodies against tyrosine hydroxylase (TH) or choline acetyltransferase (ChAT) and the same four markers as used for neurons (CB, CR, NPY, and SOM), referenced to zones of dense or sparse myelination as revealed by immunohistochemistry for myelin basic protein (MBP). Results are discussed in relation to earlier reports in rodents of a “supracapsular nucleus” of the ST (Alheid et al., 1998; Shammah-Lagnado et al., 2000; McDonald, 2003).

These microstructural details are novel and relevant to further clinical and basic research inquiries of the ST. Since the raw data are digitized and accessible to the interested reader, we have selectively included identifying details as to case and section numbers.

## Materials and Methods

### Animals and Perfusion

This investigation used tissue from seven healthy rhesus macaques, with brains already processed histologically and now publicly available in collection six of MBR.^1^ The sample consisted of one newborn brain [B64, postnatal day 7 (PD7)], four infant brains (B61, B63, B65, B66, 2.5 months old), and two adult brains (B72 and B79, 8.1, and 9.8 years old, respectively). As part of experiments unrelated to the present investigation, the adult monkeys had been used for breeding, were sometimes injected with BrdU during pregnancy, and underwent C-sections as needed (see Table 1 for further details).

**TABLE 1:** Animals listed by age of sacrifice (SAC).

MBR brain	Sex	Weight at birth (g)	Age at SAC	Body weight at SAC (g)	Brain weight at SAC (g)	Birth	Treatment(s)
B64	F	666	PD7	640	53.4	C-section	None
B66	F	610	PD75	1000	78.2	Natural	None
B61	M	460	PD75	974	74.0	Natural	None
B63	F	530	PD77	970	86.6	Natural	None
B65	M	520	PD78	980	79.7	Natural	None
B72	F	NA	8.07 years	8.72 Kg	90.1	Unknown	Breeding
B79	F	NA	9.80 years	7.68 Kg	90.3	Unknown	Breeding

*PD, postnatal day. Adults were used in breeding, were therefore several times pregnant, and were sometimes injected with BrdU during pregnancy. Some of the pregnancies ended by C-section.*

No animals were sacrificed in single use for the present investigation. All animal experiments contributing tissue that eventually became part of MBR were strictly carried out according to Yale University IACUC approved protocols. In brief, animals were first anesthetized with an intramuscular injection of ketamine/atropine (10 mg/0.01 mg per kg BW). After IV catheterization and induction of a deep anesthetic state with sodium pentobarbital (25–50 mg/kg), monkeys were transcardially perfused with ∼500 mls of phosphate-buffered saline (PBS, 0.9% NaCl in 0.1 M phosphate buffer) followed by ∼2,000 mls (for newborn and infants) or ∼3,000 mls (for adults) of 4% paraformaldehyde (PF) in PBS. In the case of B72, the fixative contained 0.05% glutaraldehyde as well. All brains were removed and postfixed overnight at 4^o^C in 4% PF in PBS. The hemispheres were separated at the midsagittal plane and immersed in 20% and then 30% sucrose (in PBS) until they sank.

### Immunohistochemistry

Tissue processing was done either in-house as previously described (Duque et al., 2007, 2013) or by FD NeuroTechnologies, Inc. (Ellicott City, MD, United States) as per MBR specifications. All processing steps, independent of processing site, were identical. In the latter case, after sucrose cryoprotection, one brain hemisphere was sent to FD NeuroTechnologies *via* overnight priority shipping at room temperature (RT), wrapped in gauze (non-woven 4” × 4” sponges), and sealed in a 250-ml Nalgene jar filled with cold 20–30% sucrose (in PBS). Brain hemispheres were cut in the coronal plane at 50 μm on a freezing microtome and sections stored in freezing solution (25% glycerol and 30% ethylene glycol by volume in PB, or FD tissue cryoprotection solution™) until further processing.

For immunohistochemistry, all rinses and incubations were done on free-floating sections (all, from the right hemisphere) in 0.1 M PBS and at RT unless otherwise stated. Stored and cryo-protected sections were thoroughly rinsed to remove the cryoprotectant solution and subsequently immersed in 0.2–0.5% hydrogen peroxidase (5–10 min) to inactivate endogenous peroxides. Parallel series of sections spaced 1 mm apart (i.e., one series repeating every 20 sections) were processed for the same antibody (Table 2). For this, sections were incubated, with gentle agitation, for 48–72 h in a solution containing the primary antibody (see Table 2) and 0.1–0.2% Triton-X. After rinsing, sections were incubated for 12–24 h in secondary biotinylated antibody, at 1:200–500 dilution with 2–5% serum from the animal host of the secondary antibody (or bovine serum albumin), with a final incubation in avidin–biotin–peroxidase complex (Vectastain elite ABC kit, Vector Labs, Burlingame, CA, United States; 4 h). Label was visualized by 0.05% 3′,3′-diaminobenzidine (DAB) as a chromogen, precipitated by 0.01% hydrogen peroxide. After three- to five thorough washes, all sections were mounted on glass microscope slides, dehydrated in ethanol, cleared in xylene, and coverslipped with Permount^®^ (Fisher Scientific, Fair Lawn, NJ, United States). DAB immunoreacted sections were stored at RT.

**TABLE 2:** Primary antibodies.

Antibody	Description	Catalog #	RRID	Company	Dilution
CB	Monoclonal mouse anti-CB-D-28K; clone CB-955	C9848	AB_476894	Millipore-Sigma	1:1,000
ChAT	Monoclonal mouse anti-ChAT; clone CL3173	NBP2-46620	**	Novus	1:2,000
MBP	Monoclonal rat anti-MBP (82–87); clone 12	MAB386	AB_141607_94975	Millipore-Sigma	1:500
NeuN	Monoclonal mouse anti-NeuN; clone A60	MAB377	AB_2298772	Millipore-Sigma	1:3,000
PV	Monoclonal Mouse anti-PV; clone PARV-19	P3088	AB_141607_477329	Millipore-Sigma	1:2,500
CR	Polyclonal rabbit anti-CR	AB5054*	AB_141607_2068506	Millipore-Sigma	1:20,000
NPY	Polyclonal rabbit anti-NPY	N9528	AB_141607_260814	Millipore-Sigma	1:8,000
TH	Polyclonal rabbit anti-TH	P40101	AB_2313713	Pelfreeze	1:500
SOM	Polyclonal sheep anti-SOM	CR2056SP	AB_141607_1288773	Fitzgerald	1:3,000
5HT	Polyclonal rabbit anti-5-HT	20080	AB_141607_10718516	ImmunoStar	1:10,000

*This antibody was discontinued by the vendor. **This antibody has not been registered in the Research Resource Identifier (RRID) system.

### Microscopy: Scanning and Posting

Sections were scanned with a 20× objective, using an Aperio CS2 HR scanner (Leica Corp). Digitized images were loaded and organized into Leica’s Aperio eSlide Manager.

Galleries of zoomable and downloadable images were made for series of sections corresponding to the same brain and same label and uploaded into MBR Collection 6 following MBR established protocols.

### Region of Interest and Cell Counts

**FIGURE 1:**
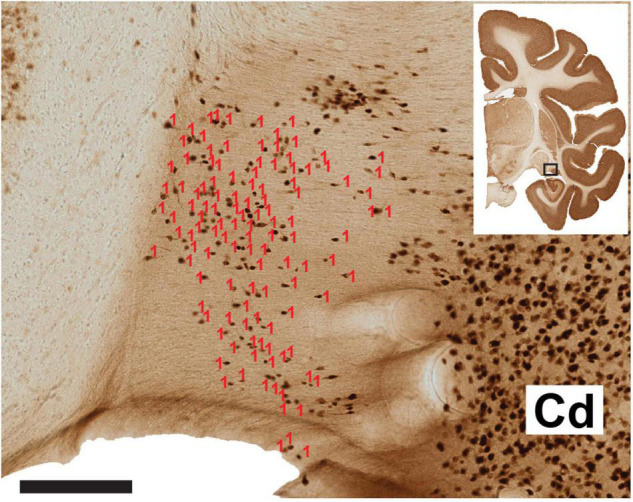
Representative section of the ventral ST and laterally adjoining caudate nucleus (Cd). The number of neurons (visualized by NeuN) has been scored in Image J software (red counters). A dorsal cell cluster was not counted, as this was considered ambiguous or comprised of neurons from the Cd, as judged by reference to an adjoining ChAT reacted section (not shown). The coronal section micrograph at upper right indicates the AP coronal level, with the hollow box highlighting the vST and the adjoining caudate nucleus. Scale bar = 200 μm. From B63 NeuN section 38.

**FIGURE 2:**
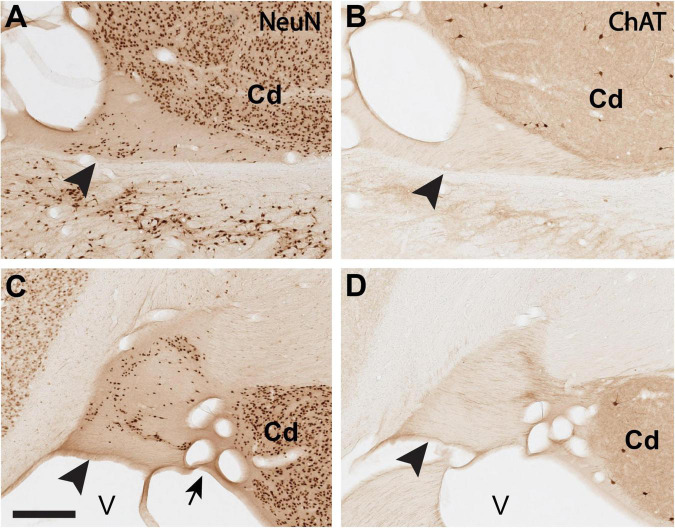
Delineation of the ST-Cd border is aided by comparison of immuno-labeling for NeuN **(A,C)** and ChAT **(B,D)**, where the ST is comparatively poor in ChAT. **(A,B)**: Closely adjoining coronal sections (<1.0 mm apart) of the dST (arrowheads) reacted for NeuN **(A)** or ChAT **(B)**. **(C,D)**: Closely adjoining sections (<1.0 mm apart) of the vST (arrowheads) reacted for NeuN **(C)** or ChAT **(D)**. Medial is to the left. Arrow in panel **(C)** points to a cluster of blood vessels, cut in cross section. Cd, caudate; V, lateral ventricle. Scale bar, 400 μm. For reference, panel A corresponds to B61 NeuN section 35. Panel **(C)** corresponds to B61 NeuN section 34.

For the sake of better cross-brain comparisons and standardization, we set a subtotal region of interest (ROI) of the ST from the anterior portion of the lateral geniculate nucleus (LGN) to the posteriormost LGN (about 7.0 mm). More anteriorly, the vST becomes less distinct as it approaches and merges into the amygdala. The posterior extent, where the dorsal and ventral ST (dST, vST) sectors join adjacent to the lateral ventricle, is also avoided. Within the designated ROI, labeled somata were manually counted in the single image plane. Each image was imported into FIJI,^2^ and labeled cells were scored by the “cell counter” plugin (Figure 1). Somata that were out of the optimal focus plane or otherwise ambiguous were excluded from the counts. In each coronal section, ST borders were defined with reference to adjacent sections reacted for ChAT, where the presence or absence of ChAT + fibers helped distinguish the ST proper from the adjoining, and possibly confounding, caudate nucleus (Figure 2). Cells touching the inclusion/exclusion borders of the ST were included. Raw data counts were analyzed across the AP extent of the defined ROI per brain and per age category.

The cross-sectional areas of the dST and vST were quantified in a sampling of six NeuN-stained sections per brain, using the pen tool of Aperio ImageScope (v.12.4.3.5008, Leica Biosystems) to draw borders at high magnification from the digitized scans. The larger blood vessels at the edge of the ST (mostly lateral for vST and medial for dST) were avoided while drawing the ROI perimeter. ImageScope provides the corresponding areas in μm^2^.

### Statistics

Due to small sample size [*n* = 1 (PD7), n = 4 (2.5 months), and n = 2 (adults)], we did not undertake extensive statistical analysis. Quantitative measures are provided in Figure 5 and Tables 3, 4.

**TABLE 3:** Number of plotted NeuN positive cells in the dorsal and ventral ST, at a sampling rate of 1/20 sections (=1.0 mm spacing).

Section	B64 ♀ PD7	B61 ♂ infant	B63 ♀ infant	B65 ♂ infant	B66 ♀ infant	B72 ♀ adult	B79 ♀ adult
**Dorsal**							

1	144	161	125	222	227	73	306
2	72	95	114	188	97	78	94
3	127	81	73	90	70	55	107
4	124	93	117	67	264	42	129
5	91	105	66	137	70	74	93
6	101	127	131	97	40	51	82
Mean	109.8	110.3	104.3	133.5	128	84.7	135.2
SD	26.6	29.2	27.7	69.8	93.5	45.1	85.2
Est. mean	2,196	2,206	2,086	2,670	2,560	1,694	2,704

**Ventral**							

1	90	200	125	107	102	60	162
2	56	44	37	52	106	60	154
3	62	67	63	38	45	24	78
4	63	65	89	62	74	23	43
5	43	67	86	54	74	26	43
6	63	82	69	63	89	45	40
Mean	62.8	87.5	78.2	62.7	81.7	39.7	86.7
SD	15.4	56.4	29.6	23.5	22.5	17.7	57.1
Est. mean	1,256	1,750	1,564	1,254	1,634	794	1,734

*Section 1 is anterior in the series. The mean number of NeuN + cells per section, times 20 (i.e., section-to-section within-series gap), gives an estimated (Est.) mean. Note that this is in the thousands of cells just in the delimited ROI.*

**TABLE 4:** Cross-sectional areas of the dorsal stria terminalis (dST) and ventral stria terminalis (vST) measured in six sections per brain.

Coronal cross-sectional area (μm^2^) of the ST (using NeuN) in the ROI						
B64 ♀ PD7	sec36	sec37	sec38	sec39	sec40	sec41
Area-dorsal	194173.35	192158.44	156049.53	190781.49	200163.1	244816.51
Area-ventral	208156.18	188514.86	199147.44	183533.26	189250.42	186388.09
B66 ♀ infant	sec36	sec37	sec38	sec39	sec40	sec41
Area-dorsal	277953.52	229368.92	312524.87	293424.27	233562.01	336410.83
Area-ventral	254953.03	244509.69	254602.56	229854.16	295142.41	391598.70
B63 ♀ infant	sec37	sec38	sec39	sec40	sec41	sec42
Area-dorsal	420311.89	268077.50	269351.24	276506.95	265774.21	313439.11
Area-ventral	465144.61	349847.46	254636.79	262994.73	299051.59	277273.06
B61 ♂ infant	sec34	sec35	sec36	sec37	sec38	sec39
Area-dorsal	358518.97	267571.26	244433.97	227167.44	289252.18	269005.09
Area-ventral	365961.01	347185.43	292520.35	285218.54	254711.62	251321.79
B65 ♂ infant	sec35	sec36	sec37	sec38	sec39	sec40
Area-dorsal	381877.43	267062.85	280521.24	323246.41	325602.76	302323.83
Area-ventral	326754.08	266164.14	253140.21	242251.46	235045.61	260411.47
B72 ♀ adult	sec38	sec39	sec40	sec41	sec42	sec43
Area-dorsal	231220.04	256074.58	273245.40	290996.91	282028.64	330878.71
Area-ventral	489413.75	360459.94	354809.59	346450.76	354974.39	358406.98
B79 ♀ adult	sec37	sec38	sec39	sec40	sec41	sec42
Area-dorsal	223861.48	322628.05	368572.64	362161.91	336928.02	397617.47
Area-ventral	549882.03	456895.23	437682.14	458538.92	424323.51	396406.97

*The sections correspond to those stained for NeuN and span the ROI anterior to posterior. For corroboration, measurements were compared to six corresponding sections stained for ChAT in three of the brains: B63 dST and vST, B61 vST, and B79 dST. The largest difference in the mean measured area was for B63 dST and corresponded to 2.72%. The smallest difference was for B63 vST and corresponded to 0.18%.*

## Results

**FIGURE 3:**
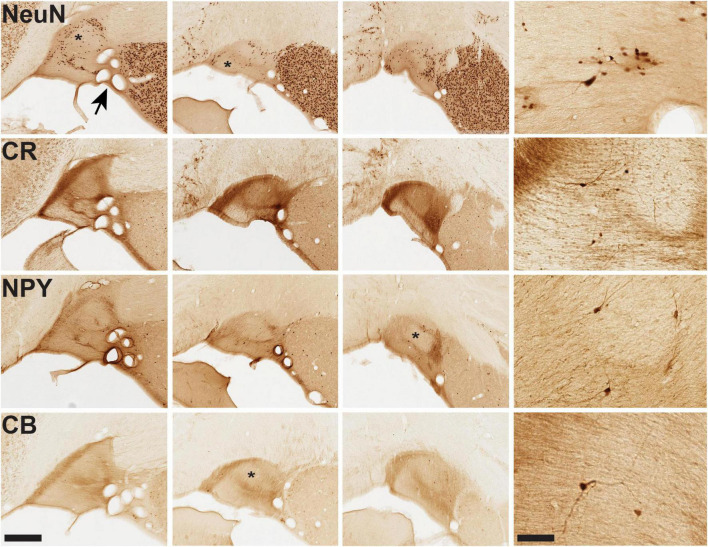
Microstructural features of vST from infant brain B61. For the four markers shown, closely adjoining sections are illustrated from three AP coronal levels, anterior (at left) to posterior (at right). Immunohistochemistry (IHC) for NeuN demonstrates pan-neuronal distribution, while IHC for CR, NPY, and CB reveals smaller subpopulations of neurons together with neuropil. Distinct fiber bundles are evident at the medial edge of vST (at the left). Conspicuous “hollows,” as visualized by these markers, are indicated by an asterisk in several of the images. Arrow at upper left image (NeuN) points to a cluster of coronally sectioned blood vessels, visible throughout. Representative fields of neurons are illustrated for each marker in the higher magnification panel at the far right. Scale bars = 400 (left) and 50 μm (right).

**FIGURE 4:**
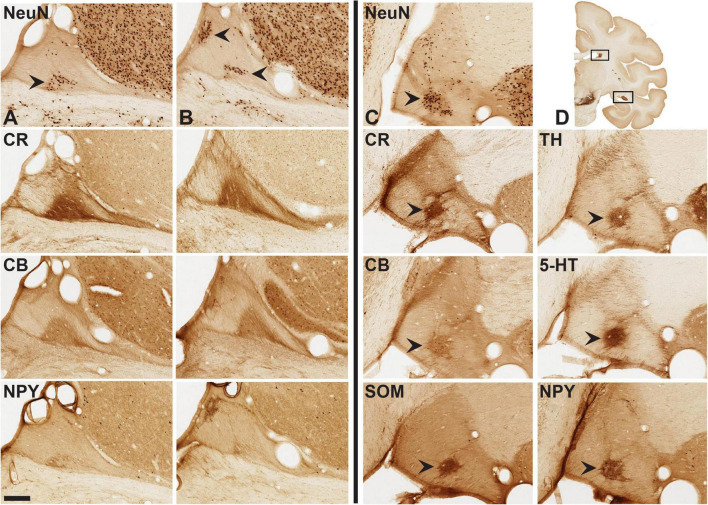
Aggregates of neurons [arrowheads in **(A–C)**: NeuN] and mixed neurons/neuropil (CR, CB, NPY). Panels **(A,B)** are two closely adjoining AP levels of dST, with three further sections in the subjacent rows. Sections at left of vertical line are from adult brain 72, and those at right of the vertical line are from adult brain 79. Arrowheads in brain 79 (at right) indicate cell, and/or neuropil aggregates in closely adjoining sections for CR, CB, SOM, TH, 5-HT, and NPY. Coronal section inset **(D)** indicates general location of dST and vST (hollow boxes). Medial is to the left. Scale bar = 200 μm.

At anterior levels of our ROI, the vST is typically shaped as a dorso-ventrally elongated rectangle, which shifts with posterior progression to resemble something like a haystack (Figure 3). More posteriorly yet, it is a dorso-ventrally elongated oval (i.e., sectioned obliquely in the coronal plane) before joining with the dST and closing the three-dimensional “C-shape” in a periventricular position (see MBR Collection 6). This configuration was consistent, except that in one of the infant brains (B65), the vST had, instead, a predominantly horizontal orientation (but not at the most anterior portion). For the same ROI, the dST is a mediolaterally (ML) oriented thick crescent or squat triangle with a wide ventral base, bordered medially by the lateral ventricle and laterally by the caudate nucleus (Figures 2, 4).

**FIGURE 5:**
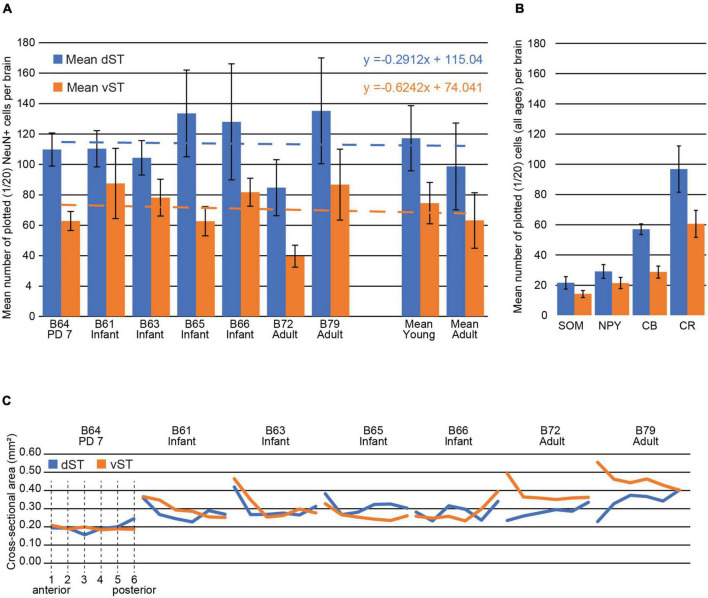
Stria terminalis (ST) cell numbers and ST size (and see Tables 3, 4). **(A)** Mean number of plotted NeuN + cells in the dST (blue) vs. vST (orange) for one early postnatal brain (P7), four infant brains (2.5 months old), and two adults. The P7 and infant brains have higher average cell counts (see mean numbers at right), and dST counts were consistently higher than vST across all brains. Note that the ratio of the mean number of plotted NeuN + cells in vST vs. dST was strikingly similar in both young and adult brains: The mean number in the vST is 64% of that in the dST. The mean percent decrease in the number of cells from young to adult in both the dST and vST was 15%, but this measure is heavily skewed by B72. Best fit lines for the mean dST and vST are almost parallel indicating a high degree of confidence in this measure. Error bars are SEM. **(B)** The mean number of plotted, immunohistochemically identified cells for SOM, NPY, CB, and CR for all brains, at a sampling rate of 1/20 sections. With the numbers in the dST representing 100%, the corresponding ratios in the vST are 65.6% (SOM), 73.5% (NPY), 50.4% (CB), and 62.5% (CR). The most numerous subpopulation is that positive for CR and the least numerous, the subpopulation positive for SOM. **(C)** The dST and vST areas, measured in NeuN-stained material, are shown from the anteriormost section (1) to the posteriormost section in the ROI (6) for each of the brains. Areas here are provided in mm^2^ for convenience (see Table 4 for further details).

### Numbers of Cells

The pan-neuronal marker NeuN shows scattered cell bodies in the dST and vST, in the newborn (PD7), infant, and adult brains (Figures 2–4 and Collection 6, “NeuN”). As seen in the figures (and: website Collection 6), there is a surprisingly high number of neurons in the ST (see Table 3). These are more numerous in dST than vST (Figure 5 and Table 3). Despite a relatively high degree of inter-brain variability (e.g., adult monkey B72), there is otherwise only a slight age-related trend, with infant brains tending to have higher average cell counts than the two adult brains. Notably, we saw no significant difference between the PD7 brain (*n* = 1) and the four 2.5-month-old brains. Across the AP extent of our ROI, there was a slight trend (Table 3) for more cells at anterior levels, but no discernible further trend with posterior progression (over six sections; i.e., a 6.0 mm extent). Neuronal subpopulations were visualized by immunocytochemistry for CR, CB, NPY, and SOM. Of these, the SOM + was the least numerous and the CR + neurons were the most numerous in the newborn, infant, and adult brains, for both vST and dST (Figures 3–5). These neurons are likely to be GABAergic but GABA immunocytochemistry, although showing expected GABergic populations in the reticular thalamic nucleus and other structures, has been less successful in our hands for the ST.

### Patterns of Cells

**FIGURE 6:**
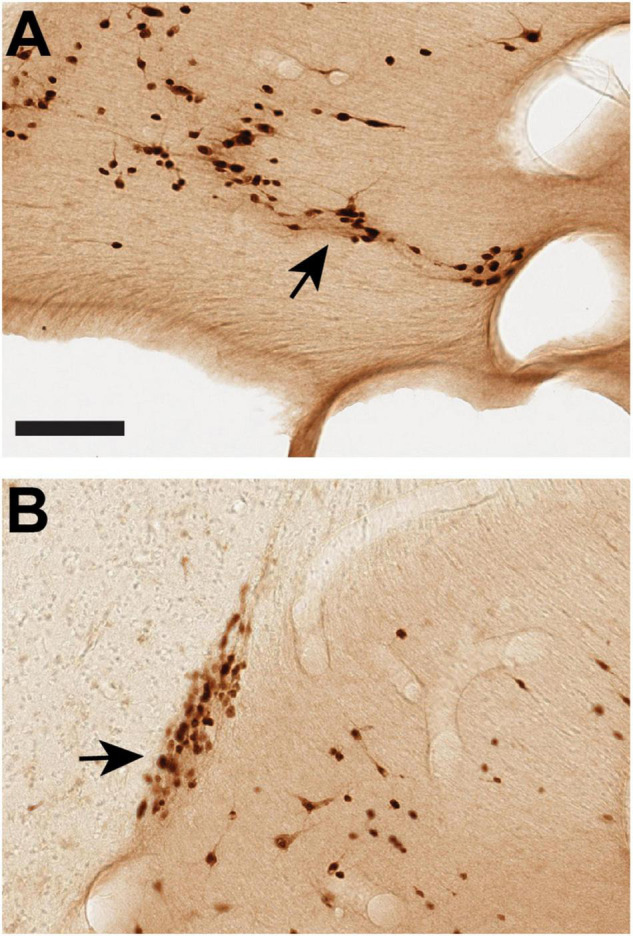
Cells within the stria terminalis (ST) can associate with (”coat”) blood vessels, as shown by two examples from vST (arrows) processed for NeuN in B61 section 34 **(A)** and B65 section 35 **(B)**. Medial is to the left. Scale bar = 100 μm.

In the newborn, infant, and adult brains, labeled cells were observed either in spatially organized patterns or were few and scattered. The most conspicuous patterns were (1) a circular or semi-circular formation, prominent in the dorsal half of vST (Figure 3), (2) a selectively elevated cell density along the medial edge of vST (Figure 3), and (3) cellular aggregates in both dST and vST (Figures 3, 4). For the dST, aggregates occurred at the medial, periventricular edge, and at the approximate mid-point of the ventral base of the ST. For vST, a ventrolateral aggregate was especially conspicuous (see for instance B72, sections 43 and 44 in NPY; B72, sections 41 and 42 in CR and CB; and B61, sections 39 and 40 in CR, CB and SOM). Both the vST (laterally) and dST (medially) are closely associated with major thalamostriatal blood vessels. In fortuitous sections, NeuN + neurons can be seen as almost “coating” segments of the smaller blood vessels (Figure 6).

### Cellular Morphology

A range of soma sizes was apparent, from tiny (<5.0 μm) to large (∼20 μm, for NPY, CB, and NeuN). Occasional somata were close to 25 μm (e.g., B61 NPY section 37 vST). Sporadic Golgi-like dendritic labeling was evident (Figure 3), but further quantification would require consecutive serial or thicker sections.

### Fiber Bundles (Ventral Stria Terminalis)

**FIGURE 7:**
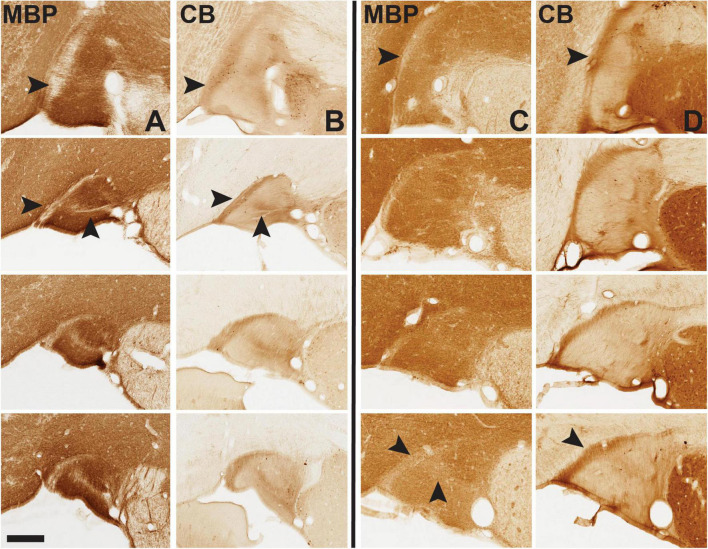
Immunohistochemistry (IHC) for myelin basic protein (MBP, **A** and **C** columns) and calbindin (CB, **B** and **D** columns) demonstrates complementary fiber patterns, shown here for vST in infant brain 61 (left of the solid black line) and adult brain 72 (at right). Anterior sections are at the top, to more posterior at the bottom of the vertical rows for MBP and CB. Note prominent MBP-sparse band at the medial edge of the ST, which coincides with a dense CB + band (arrowheads in top horizontal row). At mid and posterior levels, the band has a horizontal ML component and appears more like a “Y” (second arrowhead), most prominent in the infant and much less in the adult, especially toward posterior levels. Medial is to the left. Scale bar = 350 μm. For reference, the most posterior B61 MBP image is from section 39 of that series. For higher magnification views here (and throughout) please visit Collection 6 in MacBrainResource.

**FIGURE 8:**
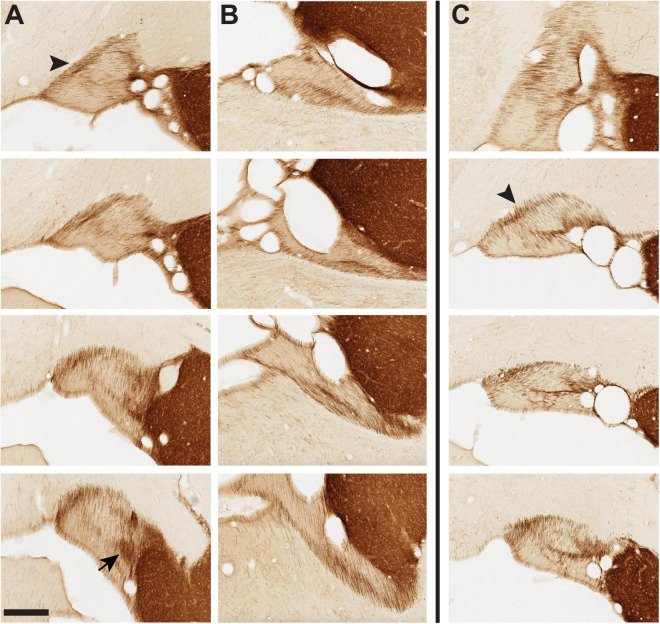
Tyrosine hydroxylase+ (TH+) fibers in panel **(A)** vST of infant brain 61, panel **(B)** dST of infant brain 61, and panel **(C)** vST of infant brain 65. Brain 61 is typical of our sample, where TH + fibers form a semi-circumferential band in the vST, especially prominent at the medial edge [arrowhead in panel **(A)**], with dorsal and ML components. The arrow at the bottom of row **(A)** indicates a concentrated neuropil aggregate at posterior levels (cf. Figure 4). **(C)** In brain 65, unusually, the gross orientation of the vST is rotated such that the medial band is now dorsal (arrowhead). A distinct pattern of TH + fibers remains evident, with a second ML band at the mid-portion of the vST. For dST in brain 65, there is an intricate fiber architecture, but dissimilar to the more typical pattern in the other three infant brains (see MacBrain data set for brain 65, sections 33–43). Anterior sections are at the top, with a posterior progression toward the bottom of each row. Medial is at the left. Scale bar = 350 μm.

Many of the histochemical markers used (CB, CR, NPY, and SOM) showed both cells, usually sparse, and fiber bundles, usually denser. Figures illustrating cellular distribution are thus also relevant for fiber bundles. As remarked in the early observations of Johnston (1923, in rodent), the ST is a complicated tract comprised of multiple axonal subpopulations. Here, we used myelin staining (MBP) as a pan-fiber label to reveal a complex micro-organization. For vST, a conspicuous myelin-poor band was readily visible at the medial edge (but see below: B65) and, less evident, a central circular zone of elevated myelin density (Figure 7). The myelin-poor medial band assumed a tilted “Y” configuration with posterior progression in our ROI. The myelin-poor zones spatially corresponded to concentrations of fibers positive for CB, CR, or TH (Figures 7, 8).

Fibers labeled by NPY or SOM were more circumferential in pattern, generally surrounding the perimeter of the vST except for the ventral-most portion. At mid- to posterior levels of our ROI, these fibers were notably denser laterally, approximately complementary to the medially biased CB+, CR+, and TH+ subpopulations. This pattern was more evident in the newborn and infant brains than in the adults.

Especially in series reacted for CR, NPY, and SOM, there was further indication of complex microstructure, both as concentrated aggregates (e.g., B72, NPY sections 37 and 43; B72, SOM section 36) and as empty, “gap” zones with sparse or no signal. These gap zones typically had a surprisingly regular circular or oval shape, were continuous across much of the AP extent of the vST, partly coincided with the myelin-dense oval zone, and were consistent across brains (for representative examples see B61, NPY sections 35, 37, and 40; SOM sections 37–40 and, anteriorly, section 33; CR sections 36 and 38). Up to five distinct compartments were delineated by smaller belts of labeled fibers (cf. B72, CR section 38). ChAT + fibers were not dense but formed a distinct dorsolaterally situated bundle (not illustrated but see MBR collection 6). In B65, there was a dense ChAT + band coinciding with the ventral branch of the myelin-poor “Y.”

### Fiber Orientation (Ventral Stria Terminalis)

Fiber orientation appeared uniform across the different subpopulations but was variable along the different AP portions. The shifts are clearest in the TH series (Figure 8). At the anterior portion of our ROI, TH fibers have a marked ML orientation, which becomes (in the coronal plane of section) dorsoventral at posterior levels. In between, there are two further shifts of orientation. First, from coursing ML, fibers take a pronounced dorsolateral to ventromedial (2:00 to 7:00) orientation. Then, in a further shift, the orientation changes to dorsomedial and ventrolateral (11:00–5:00).

In B65, where the vST had an unusual gross anatomical horizontal orientation, fiber compartments were still apparent, but with some modifications (Figure 8). Again, taking the MBP series as reference, there were noticeable MBP zones, but these were displaced to the dorsal (not medial) edge, and what could be a myelin-poor tilted “Y” (as in B61) was shifted 90° and then inverted (i.e., the open Y points laterally in B65, but medially in B61). In this brain, the CB+, CR+, and TH+ fiber pattern was overall similar and generally complementary to that of MBP, that is, corresponding in location to MBP-poor regions.

### Fiber Bundles (Dorsal Stria Terminalis)

In three of the four infant brains, MBP, CB, and CR stains showed a bandlike pattern, with bands oriented ML. At posterior levels, the pattern became more patch-like (CB+ and CR+). One of two prominent patches was located at the medial edge of the triangular ST, and a second about midway along the ventral base (Figure 4). The pattern in the adult brains was more patch-like throughout. Consistent with our observations for vST, zones of dense CB+, CR+, and TH+ fibers appeared to be coincident together, but complementary with myelin-poor zones. The distribution of NPY+ and SOM+ fibers at anterior levels was patchy, and circular or oval “empty” zones were common in both NPY and SOM.

For B65, the pattern of CB+, CR+, and TH+ fibers was generally similar in the dST, all three seemingly complementary to myelin-poor zones. The overall organization for all markers appeared less bandlike and more patchy in this brain with discontinuous aggregations and hollows (e.g., sections 33, 35, 37, 39, 41, and 43).

### Fiber Orientation (Dorsal Stria Terminalis)

In the newborn and infant brains, at anterior levels (e.g., B61, B65 TH section 33), fiber bundles in the coronal plane had a laterodorsal to medioventral orientation (2:00/7:00). About 4.0 mm posterior, this shifted to ML (B61, B65 section 37) and then, with further posterior progression shifted again, from mediodorsal to lateroventral (11:00/5:00). This is the same progression observed for the vST. In the dST of the adult brains, however, fibers at anterior levels were oriented predominantly ML (TH sections 36–38), before shifting toward a more mediodorsal to lateroventral slant (for example, TH section 39). The “outlier” infant brain (B65) had the same orientation shifts as the other young brains, unlike the situation for the vST.

### Cross-Sectional Area of Stria Terminalis

Since the cross-sectional size of the ST is relevant for evaluating cell density, we measured the mean cross-sectional areas of the dST and vST in six sections of our designated ROI (Figure 5C and Table 4). The smallest areas were in the PD7 animal and the largest in the adults, as might be expected. In the young animals, the relative size of the dST or vST fluctuated at different AP levels. However, for the adults, throughout the AP extent of the ROI, the vST was always larger than the corresponding dST; the cross-sectional area of the vST was decreasing in the AP direction, while that of the dST was increasing (Figure 5C). Thus, on average, across all brains, the density of NeuN + cells in the dST was 415 ± 239 (SD) and that in the vST 241 ± 110. Overall, the corresponding largest cell densities were in B64 (PD7) and the lowest densities in B72 (adult).

## Discussion

In this study, we report (1) small neuronal clusters and scattered neurons, visualized by NeuN and other cellular markers, across the ST and (2) a myelin-related fiber topology, consisting of distinct, reproducible myelin-poor zones across individual brains that correlate with other neurochemical markers. The cellular aggregates recall the “pockets” extensively investigated within the rodent ST and associated with the idea of an “extended amygdala” (Alheid et al., 1998; Shammah-Lagnado et al., 2000). In NHP, as in rats (McDonald, 2003), the cell aggregates (”columns”) are discontinuous and intermingled with more scattered neurons. Demonstrating the full three-dimensional organization would require uninterrupted serial section or en bloc visualization.

### Cellular Organization

Neurons within the ST have been reported by numerous studies in rodents and NHP (Martin et al., 1991; Alheid et al., 1998; Shammah-Lagnado et al., 2000; McDonald, 2003; and references therein). These might be intrinsic interneurons, extrinsically projecting neurons, or neurons intruding from adjacent structures, in this case, the laterally adjacent caudate nucleus. Of these possibilities, there is evidence that at least some of these neurons are extrinsically projecting; namely, in rats, a subpopulation of NPY+ neurons scattered throughout the AP extent of the ST is retrogradely labeled by tracer injections in the basolateral amygdala (Leitermann et al., 2016). In addition, afferent and efferent connections of the neurons within the ST (”supracapsular component”) have been extensively mapped in the rodent brain by anterograde and retrograde tracers (Shammah-Lagnado et al., 2000; McDonald, 2003).

Given that the ST is immediately adjacent to the caudate nucleus, we cannot eliminate the possibility that some striatal neurons may have intruded into the ST. The ambiguous border between the ST and caudate nucleus has been remarked in rodents (Alheid et al., 1998), with the comment that the cells in the “lateral pocket” of the supracapsular ST were indistinguishable from the medium-sized striatal cells. de Olmos and Heimer (1999) further commented on an “interface island,” between the ST and adjoining caudate. For this reason, our plotting of ST cells was conservative in avoiding those closer to the border with the caudate nucleus.

Other white matter (WM) environments contain neurons. The fornix, as a defined and relatively isolated tract, offers an obvious comparison to the ST. Overall, from our observations in MBR, there are very few or no neurons in the ventral fornix; but in the dorsal fornix of newborn, infant, and adult, there are scattered neurons and small neuronal clusters, especially at or slightly off the midline (e.g., see NeuN in B72, sections 36–44, and in B61, sections 33–42). Neurons are also detected within the anterior commissure. Rapid-Golgi preparations of the anterior commissure in other studies demonstrate both interneurons and projection neurons with axon collaterals [in rats: Larriva-Sahd et al. (2002)]; and immunohistochemistry reveals reelin + neurons that are positive for GABA (Misaki et al., 2004). White matter (WM) neurons (aka “interstitial neurons”) are a constant feature in the corpus callosum [NHP: Rockland and Nayyar (2012); human: Jovanov-Milosevic et al. (2010)] and in the superficial and deeper subcortical WM (Mortazavi et al., 2016; Cebada-Sanchez et al., 2018; Sedmak and Judas, 2021; and references therein).

The functional significance of WM neurons remains unclear and is likely to be diverse. In early development, they are likely to play a role in axon guidance [mouse: Niquille et al. (2009); human: Jovanov-Milosevic et al. (2010) and Culjat and Milosevic (2019)]. In the adult, they presumably participate in regulatory mechanisms. A small subpopulation of cortical WM neurons, positive for nitric oxide, sends long-distance projections to gray matter cortical targets [in macaque: Tomioka and Rockland (2007)]; and, in the corpus callosum, neurons expressing NADPH diaphorase occur in close association with blood vessels (Rockland and Nayyar, 2012). The suggested role in vascular regulation may apply to neurons in the ST, where these can sometimes be seen in close association with blood vessels (Figure 6).

Subcortical WM neurons are more abundant in the infant brain [Kostovic and Rakic (1980); and see examples in Collection 6]. In our relatively small sample, one but only one of the adult brains (B72) had fewer neurons than any of the four infant brains. The possibility of a developmental gradient was not supported by our single PD7 brain (B64), which showed no significant numerical difference with the 2.5-month-old brains. In our limited sample, the ratio of the mean number of NeuN + cells in vST vs. dST was strikingly similar. The number in the vST consistently is 64% of that in the dST, in both young and adult, perhaps indicating that the proportion of cells in the different compartments of the ST is conserved from birth to adulthood (Figure 5). By subpopulations, there was some fall off in CR, NPY, and SOM numbers in the adult. Although there was a high degree of inter-individual variability, neuron numbers were consistently greater for the dST than the vST (Figure 5 and Table 3, averaged over six sections).

### Fiber Bundles

Our results demonstrate a complex fiber microarchitecture in the ST, as globally visualized by myelin stains. In the vST, there is a central circular zone of denser myelination, and at the medial edge of the vST, a prominent myelin-poor zone which morphs to a “tilted Y” with posterior progression. Of the substances screened here, zones of heightened CB, CR, and TH were concordant with the myelin-poor zones; but fibers positive for NPY or SOM formed more laterally biased, circumferential bundles without a clear relationship to myelination. Previous studies have illustrated a medially situated CB + fiber band (Pitkanen and Amaral, 1993; their Figure 17); and a mix of myelinated and unmyelinated fibers has previously been reported at electron microscopic resolution in the ST of rats (Alheid et al., 1998) and in the ventral amygdalofugal pathway in NHP (Novotny, 1977). Finer organization, as we describe in the Results (and Figure 8), is prominent in tissue reacted for TH.

The ST is often described as a bidirectional tract with axons running between the amygdala and BNST, although monoaminergic and other fiber systems are known to be included [rodent: Alheid et al. (1998) and Shammah-Lagnado et al. (2000); NHP: Martin et al. (1991)]. The neurochemical subpopulations reported here are consistent with an origin from the complex neuronal subpopulations in the amygdala and BNST (Beyeler and Dabrowska, 2020; Hammack et al., 2021; McDonald, 2021). The specific neuronal identity and origin have not yet been mapped, although the medially situated CB + bundle was thought to originate from large type 4 neurons in the basolateral amygdala [in NHP: Pitkanen and Amaral (1993)].

Some degree of topographic organization in the ST is likely; for example, for amygdalostriatal fibers [see anterograde tracer injections, in Russchen et al. (1985); their Figures 2, 4, 8]. For comparison, in the relatively well-investigated fornix, a series of tracer placements in different monkeys showed an orderly topographic organization of fibers originating from different parts of the subiculum, presubiculum, and entorhinal cortex (Saunders and Aggleton, 2007).

The gross anatomical rotation of the ST and rearrangement of the microstructure in B65 were pronounced. In this case, we note that this monkey was born 5 days premature [although of adequate body weight (520 g)]. Whether this fact or known subsequent postnatal stress may have been a factor in the rearrangement (either causative or correlative of something else) is not known.

## Conclusion

In this report, we describe an intricate cellular and fiber microstructure of the ST in the newborn, infant, and adult macaque brain. Multiple questions remain for further investigation, including a more extensive developmental time series, quantitative comparisons of cellular components across the full ST trajectory, and a more detailed co-mapping of cellular and axonal architectures. Double label in the same section for NeuN and GABA is needed to confirm whether all or most of the neurons are GABAergic, as might be expected given the preponderance of GABAergic neurons in the central and medial amygdala and BNST. This complex neuropil is phylogenetically conserved over rodents and NHP. It can be assumed to have multiple functional roles, probably with changes over the lifespan and with important implications for normal and pathological processes.

## Data Availability Statement

The datasets presented in this study can be found at: https://medicine.yale.edu/neuroscience/macbrain/. Further requests can be directed to the corresponding author.

## Ethics Statement

Original animal studies that produced archived materials used in the current study were reviewed and approved by Yale University IACUC.

## Author Contributions

AD organized and provided access to Collection 6 (MacBrain Resource). KR identified stria terminalis as a region of interest and with AD, supervised MS. MS conducted cellular and fiber analyses across brains and prepared the figures. All authors participated in the discussion of results (by weekly or bi-weekly ZOOM), mutually drafted the manuscript, and approved the final version of the manuscript.

## Conflict of Interest

The authors declare that the research was conducted in the absence of any commercial or financial relationships that could be construed as a potential conflict of interest.

## Publisher’s Note

All claims expressed in this article are solely those of the authors and do not necessarily represent those of their affiliated organizations, or those of the publisher, the editors and the reviewers. Any product that may be evaluated in this article, or claim that may be made by its manufacturer, is not guaranteed or endorsed by the publisher.

## Footnotes

1
https://medicine.yale.edu/neuroscience/macbrain/

2
http://imagej.nih.gov/ij/
